# Deterministic colonization arises early during the transition of soil bacteria to the phyllosphere and is shaped by plant–microbe interactions

**DOI:** 10.1186/s40168-025-02090-1

**Published:** 2025-04-22

**Authors:** Teresa Mayer, Erik Teutloff, Kerstin Unger, Pamela Lehenberger, Matthew T. Agler

**Affiliations:** 1 Plant Microbiosis Lab, Institute of Microbiology, Friedrich Schiller University, Jena, Germany; 2 Cluster of Excellence, Balance of the Microverse, Friedrich Schiller University, Jena, Germany; 3 Current affiliation: Professur Für Fachdidaktik Life Sciences, Technische Universität München, Munich, Germany; 4 Current affiliation: Department of Soil Ecology, Helmholtz Centre for Environmental Research, Halle, Germany

**Keywords:** Phyllosphere microbiome, Natural colonization, Soil-to-leaf transition, Colonization mechanisms

## Abstract

**Background:**

Upon seed germination, soil bacteria are activated to transition to the plant and eventually colonize mature tissues like leaves. These bacteria are poised to significantly influence plant health, but we know little about their colonization routes. We studied the mechanisms of the transition of soil bacteria to germinating plants and leaves using an *in-planta* isolation approach and by experimentally manipulating inoculation times. We then tested how plant-microbe-microbe interactions shape assembly mechanisms in natural soil communities by amending soil with a trackable, labeled strain of the opportunistic pathogen *Pseudomonas viridiflava* (*Pv*3D9).

**Results:**

We identified 27 diverse genera of leaf-associated bacteria that could transition alone from a few cells near a germinating plant to mature leaves, suggesting that the soil-to-leaf transition is probably important for them in nature. Indeed, when plants were only inoculated by soil after the emergence of true leaves, less diverse bacteria transitioned to mature leaves via different colonization mechanisms than when plants germinated in the presence of soil microorganisms. In particular, deterministic processes drove the colonization of phylogenetic bins dominated by *Pedobacter*, *Enterobacter*, *Stenotrophomonas*, *Janthinobacterium*, *Pseudomonas*, and *Chryseobacterium* only in the natural soil-to-leaf transition. Host genotype and soil amendments with *Pv*3D9, both of which affect host physiology, had strong effects on mainly deterministic colonization.

**Conclusions:**

Diverse bacteria transition from soil to leaves during natural colonization, resulting in characteristic diversity in healthy leaf microbiomes. The mechanisms of colonization are a mix of stochastic processes, which will be largely shaped by competition, and deterministic processes which are more responsive to factors that shape host physiology. In the chase toward targeted manipulation of microbiomes, identifying these mechanisms for a given host and environment provides important information. Developing targeted treatments, however, will require further dissection of the pathways by which host factors drive stochastic and deterministic transitions from soil to leaves.

Video Abstract

**Supplementary Information:**

The online version contains supplementary material available at 10.1186/s40168-025-02090-1.

## Background

Plant leaves are colonized by diverse microbes, including bacteria, fungi, and oomycetes. This microbiome plays a critical role in determining how plants deal with stresses like drought, disease, and herbivory [1, 2]. In particular, the bacterial component of the microbiome (the bacteriome) aids plant survival in the presence of detrimental soil microbes [3, 4] and can protect leaves against pathogen attack [5]. Soil is thought to be an important source of inoculum for all plant tissues, but only a small fraction of soil microbes will later make up a significant proportion of the plant microbiome [6]. This filtering is not surprising considering the many barriers to a successful transition of bacteria to germinating seedlings and ultimately mature leaves. Upon germination, a radicle and then cotyledons emerge into the vast microbial diversity contained in the soil [7]. Growth of true leaves follows only later. Thus, for soil bacteria to reach aerial leaf tissue, they must deal with edaphic (soil) factors [8], competitively reach host tissue and establish a niche [9, 10], and either grow together with the plant to establish in or on emerging leaves [11] or invade the tissues later. These early stages are likely very important determinants of the complex transition of bacteria from soil to leaves and ultimately of plant health. However, how they play out and the processes governing them are not well understood.

Stochasticity plays major roles in microbial community assembly in diverse environments [12, 13]. In the context of the plant, stochastic colonization indicates that a niche could be occupied by many different bacteria with similar fitness. In other words, such bacteria are mostly exchangeable. Given the massively diverse soil inoculum, stochasticity could dominate early immigration processes from a complex inoculum like soil to germinating seedlings. By contrast, bacteriome structure in mature leaves is determined at least partly by deterministic factors like host selection [14], and the interaction between host factors and the environment [6]. Indeed, while the abundance of many leaf taxa is correlated to their abundance in soil, suggesting stochastic dynamics, there is extensive variation in this trend that can indicate deterministic drivers [15]. Importantly, recently developed tools can use microbiome structure data to quantify the contribution of stochastic or deterministic processes. This can be done both on the level of whole microbiomes and on the level of individual taxa, to reveal taxa that may occupy specialized niches in hosts [16].

The importance of stochastic and deterministic processes in natural colonization may also be shaped by direct or indirect interactions between bacteria [17]. In plant leaves, early arriving pathogens can alter the immune state of the host and thereby facilitate the colonization of late comers [18–20]. Alternatively, early colonizers could occupy essential nutrients like iron [21] or other favorable niches [22]. Experiments simulating the invasion of mature leaves have shown that priority effects can strongly influence bacteriome assembly, with early colonizers persisting and influencing subsequent colonization outcomes [23]. During natural colonization from the soil, most bacterial taxa making the transition to leaves are likely to start from very low cell numbers, given the massive soil bacterial diversity and relatively low abundance in the soil of leaf-colonizing bacteria [15]. Whether or not the arrival of low-abundance soil bacteria can significantly affect colonization processes is unknown. However, this would be important because it may suggest ways to shape colonization mechanisms and community assembly trajectories.

Here, we experimentally dissected the transition of leaf-colonizing bacteria from soil to leaves to better understand how it shapes leaf bacteriomes and ultimately plant health. First, we tested whether and how diverse bacterial taxa can efficiently colonize leaves starting from extremely low levels near a germinating seedling, as a simple soil diversity model suggests they must do in soil. Next, we used three different *Arabidopsis thaliana* genotypes, including two wild genotypes isolated from Jena, Germany, and compared the assembly mechanisms of bacterial communities in mature leaves when plants germinated in inoculated soil vs. when inoculation occurs post-germination. Finally, we amended natural soil with a trackable opportunistic pathogenic bacterium to better understand how community assembly mechanisms are shaped by bacterial interactions. The results reveal new insights into the types of niches occupied by bacteria that transition from soil to leaves, which can help point the way toward their management.

## Materials and methods

### Plant materials

We utilized three *A. thaliana* wild-type genotypes: Col-0, NG2, and PB. Col-0 is a widely available model genotype of *A. thaliana*, while NG2 and PB are genotypes that were previously recovered from wild populations in Jena, Germany [14]. Near-isogenic lines of each were generated via multiple generations started from single seeds. NG2 is available from NASC (N2110865). PB is being made available there as well and is available upon request from the authors.

### In-planta isolation of leaf bacteria experiment

To obtain an overview of the diversity of bacteria that could realistically make a transition from soil to leaves, we developed an *in-planta* enrichment whereby bacteria from a natural mixed inoculum derived from wild *A. thaliana* leaves were given the chance to colonize a germinating seedling and successful ones were identified later in mature leaves. A test enrichment experiment was performed to optimize the procedure for recovery and sensitivity and that is described in the (Supplementary Methods and Results).

#### Production of inoculum from leaves

We sampled the leaves of wild plants in the spring of 2019. We collected 10 *A. thaliana* rosettes of different sizes from the wild populations NG2 and PB, located in Jena, Germany. They were washed three times with autoclaved ultrapure water to remove dirt, ground with a metal pestle, and suspended in PBS/S (PBS buffer + 0.02% Silwet L-77) by vortexing. After centrifugation (1 min, 19,000 × g), the inoculum was aliquoted, mixed with glycerol (final glycerol concentration: 21.5%), and stored at − 80 °C. For the experiment, the stock was diluted in PBS/S so that the inocula finally contained an average of 3.5 and 1.45 cells/seedling (for NG2 and PB, respectively—details on how the dilution level was obtained are in the Supplementary Methods).

#### Plant growth, inoculation, and identification of successful leaf colonizers

Seeds were surface sterilized with 2% bleach and 70% ethanol, washed three times with autoclaved ultrapure water, and vernalized for 4 days in 0.1% agarose at 4 °C in the dark. We sowed one seed in each well of a 24-well plate filled with 1 mL ½ MS + 0.2% sucrose + 1% agar. The plants were incubated in growth chambers (PolyKlima PK-520) at 24 °C/18 °C 16 h/8 h day/night cycle with 50% light intensity. Five microliters of leaf microbe extract (see bacteria inoculation density in the previous section) was inoculated as follows: NG2 or PB inocula were added directly onto the cotyledons of 3-day-old NG2, PB, or Col-0 seedlings. We also mock-inoculated control seedlings with PBS/S and did not observe contamination. Inoculated plants were grown for 2 weeks after which whole rosettes were harvested with flamed tweezers. After removing roots and any flower stems, they were transferred into deep 96-well plates and frozen at –80 °C until further processing. Next, we crushed and plated a dilution series of the rosettes to look for bacterial colonies. They were identified by DNA extraction and PCR amplification of the entire 16S rRNA gene region, followed by Sanger sequencing (see detailed supplementary methods).

### Inoculation time experiment

#### Preparation of microboxes

In total, 18 microboxes each containing 9 flow pots [24] were prepared, six for each of the three *A. thaliana* genotypes (Col-0, NG2, and PB). After they were autoclaved, opening the microboxes for inoculation and sampling was performed in a laminar flow hood. On the first day of the experiment (ID0) the flow pots were flushed with 25 mL sterile MilliQ water to wet the substrate. Seeds had been surface-sterilized with 2% bleach and 70% ethanol and were stratified in the dark for 3 days in 0.1% agarose at 4 °C. Between 7 and 10 seeds were added to each pot, and these were later thinned to 1–3 plants per pot. More detailed box preparation instructions can be found in the supplementary methods.

#### Inoculations

**Fig. 1:**
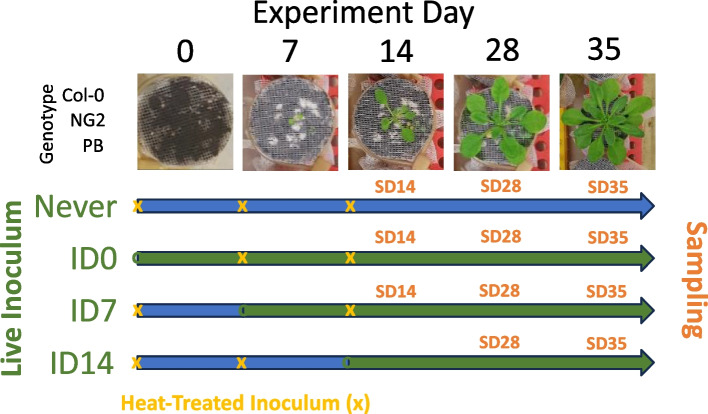
Diagram of flow pot experiment 1, showing for each of three treatments and the control the time of inoculation with live inoculum (start of green arrows), heat-killed inoculum (yellow *x*), and the sampling times (SD)

For each plant genotype, two microboxes were randomly assigned to be inoculated with a natural soil microbial community on the first day of the experiment (ID0), two microboxes on day 7 of the experiment (ID7), one microbox on day 14 of the experiment (ID14), and one microbox was only inoculated heat-killed (double-autoclaved) version of the inoculum (HK). The three inoculation times are all early in development, corresponding to the seed (D0), cotyledon (D7), and first true leaf (D14) stages of plant growth (Fig. 1). Inoculation was performed by injecting a natural soil slurry + Murashige and Skoog (MS) medium mixture into the soil via a tube attachment (5 mL of inoculum) and by spraying from above with an airbrush system (three sprays for half a second each). To ensure that treatment effects were only due to living microorganisms and not, for example, immune priming by molecular patterns, all plants that did not receive live inoculum at each treatment time received the heat-killed inoculum. The boxes were closed and sealed with medical tape (Duchefa Biochemie). Detailed information on preparing the natural soil slurry and seed preparation can be found in the supplementary methods.

#### Plant growth conditions, sampling, and processing

The prepared boxes with seeds were incubated in growth chambers (PolyKlima PK-520) at 20 °C/14 °C 16 h/8 h day/night cycle with 75% light intensity. Humidity was not controlled, but given the environment inside the closed boxes, was likely close to saturation. Plants were sampled on day 14 (SD14), day 21 (SD21), day 28 (SD28), and day 35 (SD35). At each sampling day except SD35, rosettes were collected with a flamed tweezer and placed in a screw cap tube with sterile beads (two 3-mm steel beads (WMF) and 0.2 g of 0.25–0.5 mm glass beads (Carl Roth)) and stored at − 80 °C until further processing (1 whole plant rosette = 1 sample). At SD35, only three leaves per plant were combined into one sample due to the large size of plants: an old, a middle-aged, and a young leaf. Because we wanted to focus on all leaf colonizers, we processed whole-leaf samples without separation of surface and endophytic compartments. DNA was extracted by bead-beating, followed by incubation for 10 min in a cetrimonium bromide (CTAB) buffer, direct precipitation in ethanol/sodium acetate then ethanol washing. The DNA was further purified using a magnetic bead-based cleanup. DNA extraction details can be found in the supplementary methods.

### *Pseudomonas viridiflava* 3D9 soil amendment experiment

Methods for the experimental setup to initially test how *P. viridiflava 3D9 *(*Pv*3D9) affects plant phenotypes and physiology (immune response) in natural soil are found in the supplementary methods.

#### Pv3D9-141 preparation

*Pv*3D9 was fluorescently labeled by integrating pMRE-Tn7-141 plasmid containing the mTurquoise2 gene as well as chloramphenicol and gentamicin resistance cassettes [25] to generate *Pv*3D9-141. It was grown in LB medium supplemented with gentamycin and chloramphenicol at 220 rpm and 28 °C to OD_600_ of roughly 1.5, the cells were then harvested by centrifugation for 5 min at 2000 rcf, the supernatant was removed, and the culture was washed once with 10 mM MgCl_2_. After centrifugation, the cultures were resuspended in 10 mM MgCl_2_ before diluting into the MgCl_2_ wetting solution.

#### Soil preparation and plant growth

Surface-sterilized *A. thaliana* NG2 seeds (2% bleach and 70% ethanol) were stratified in the dark for 3 days in 0.1% agarose at 4 °C. On the day of the experiment, a base soil mix was prepared by mixing 4 L Floraton 3 potting soil (Floragard), 2 L perlite (0–6 mm Perlite Perligran, Knob), and 25 g Substral Osmocote garden flower fertilizer (Celaflor). This was wetted with either 2 L of 10 mM MgCl_2_ (soil “LS”) or with 2 L of MgCl_2_ mixed with 200 g of dried garden soil (soil “LS + GS”). For the *Pv*3D9-141 treatments, cells were added to the MgCl_2_ or MgCl_2_ + soil slurry to target 1000 CFU/cm^3^ or 1,000,000 CFU/cm^3^ of soil. If the spermosphere (zone of influence) of a germinating seed (0.5 mm × 0.25 mm × 0.25 mm) reaches a distance into the surrounding soil equaling its own dimensions, then this works out to about 0.8 (“low”) and 800 (“moderate”) 3D9-141 CFUs in the spermosphere, respectively. Vernalized seeds were sown with 15 to 20 seeds per pot (5.5 cm upper diameter) and incubated at 193 μmol/(m^2^∙s) at 22 °C/18 °C and 16 h/8 h day/night conditions. To minimize batch and positional effects, the tray positions were switched randomly every 2 days.

#### Pv3D9-141 effects on shoot growth

Seven days after germination, seedling shoots were sampled by removing entire plants from the soil and cutting off the root with flamed scissors and tweezers. Because plants were very small, each replicate is the average of eight plants from one pot that were measured together. At the same time, the number of plants in one pot was thinned out to three.

#### Estimating bacterial and Pv3D9-141 loads in grown plants with CFU counting

To count bacterial loads in the leaves, 14 days after germination, six samples of two to three leaves each were collected and stored at –80 °C until they were processed. For total bacteria and 3D9-141 CFU counting, frozen leaf samples were crushed in 120 µL of 10 mM MgCl_2_ using 3-mm diameter sterilized metal beads (WMF) in a mini bead-beater 96 (Biospec Products) at 1400 strokes/min. After homogenization, a dilution series in 10 mM MgCl_2_ was drop-spotted onto three different media in duplicates and the plates were incubated at 28 °C and counts were made after 72 h (details on the media and their selection can be found in the supplementary methods). Additionally, a fluorescence stereo microscope equipped with a GFP filter (Zeiss 38 HE GFP—ex 450–490 nm/em 500–550 nm) was used to record the number of mTurquoise2 fluorescent colonies.

#### qPCR quantification of 3D9-141 leaf loads

For qPCR quantification of 3D9-141 and 16S rRNA gene amplicon sequencing, 14 days after germination, six samples of two to three leaves each were collected and stored at –80 °C until gDNA was extracted. DNA was extracted by bead-beating in a CTAB buffer, followed by a phenol–chloroform cleanup, precipitation in isopropanol, and ethanol washing. Details on DNA extraction can be found in the supplementary methods. For the qPCR, the single-copy *mTurquoise2* gene integrated into the 3D9-141 genome was targeted. For the normalization of copy numbers to the host, we targeted the *A. thaliana EF1-α* gene [26]. Copy numbers were estimated with standard curves generated from a 1:8 dilution series of the linearized pMRE-Tn7-141 plasmid and a 1:8 dilution series of NG2 gDNA. Details on the primers, reaction components, controls, and thermocycler setup are provided in the supplementary methods.

### *Bacterial community characterization *via* 16S rRNA gene amplicon sequencing*

#### Library preparation

The extracted gDNA was diluted 1:10 in 10 mM Tris–HCl (pH = 8) and used as a template for 16S rRNA gene amplification. The amplicon sequencing library preparation targeted the V3-V4 region of the 16S rRNA gene and used blocking oligonucleotides to avoid plant plastid amplification, as previously described [27] with a few modifications (see detailed steps in supplementary methods). The final library was spiked with 10% PhiX genomic DNA to ensure high enough sequence diversity and was loaded onto an Illumina MiSeq and sequenced for 2 × 300 cycles. Amplicon sequencing data was split on indices and adapter sequences were trimmed using Cutadapt 1.2.1 [28]. The quality of the reverse reads was not satisfactory, so we proceeded with high-quality forward reads only, which were clustered into amplicon sequencing variants (ASVs) using dada2 [29]. First, reads were trimmed and filtered to remove reads with more than three errors per 100 bp. Sequences were then dereplicated and denoised using the error rate information before calling amplicon sequence variants (ASVs). Chimeric ASVs were removed, and taxonomy was assigned using the latest Silva 16S rRNA gene database (v. 138.1) [30].

#### Microbial diversity analysis

Downstream analysis was performed in R with phyloseq [31] and other packages listed below. Host-derived reads were removed by filtering any ASVs in the orders “Chloroplast” and “Ricketsialles” from the 16S ASV tables and data was filtered to remove samples with less than 500 reads. For further processing, the reads were either not grouped (ASV level) or grouped at the genus and order levels to provide different robust levels of resolution. Beta diversity was based on the Aitchison distance, and the “RDA” function was used to perform either unconstrained principal components analysis on the Aitchison distance matrix or a redundancy analysis on the matrix constrained by experimental factors, as stated in the text and figures. The statistical significance of factors was tested with an ADONIS test (a permutational analysis of variance) with 999 permutations. Beta dispersion (between-sample beta diversity) was also based on Aitchison distance with statistics as described in the figures. The alpha diversity metrics Chao1 (estimate total richness of community) and Shannon (index accounting for both richness and evenness) were used, and statistical analysis was based on a non-parametric Kruskal–Wallis test. In the inoculation time experimental results, to identify taxa whose abundance in mature leaves significantly varied with experimental variables, we used DESeq2 [32] on data from combined sampling days 21, 28, and 35. The log-transformed abundances of taxa identified by DESeq were plotted in boxplots for visual inspection with a *p*-value based on a non-parametric Kruskal–Wallis test. In the *Pv*3D9-141 amendment experiment, we used the RDA axes constrained by treatment and bi-plot data to identify the taxa that were most correlated to the *Pv*3D9-141 treatments. These taxa were selected for visualization in the biplot and in the boxplots.

For the analysis of processes driving colonization, we employed the tool “Infer Community Assembly Mechanisms by Phylogenetic bin-based null model analysis” (iCAMP 1.5.12) [16]. First, we generated a phylogenetic tree. The 16S rRNA sequences of the ASVs were aligned using the DECIPHER (2.20.0) function AlignSeqs and highly variable regions were masked using MaskAlignment. The masked alignment was then converted to the phangorn package (2.11.1) with the function phyDat and output as a fasta alignment with the function write.phyDat [33]. Finally, the alignment was fed to RAxML next generation (1.2.1) to generate a phylogenetic tree using the GTR + G + I model with default parameters (20 tree searches with 10 random and 10 parsimony-based starting trees) [34]. iCAMP was run by loading the OTU table, taxonomy information, and tree and generating a treatment table that linked the samples to the plant ecotype and inoculation day. For the environment table, the ecotype information was provided. To optimize parameters, first, the phylogenetic niche preference was assessed and then the within-bin phylogenetic signal was measured. We tested a range of values for ds and bin.size.limit and chose those where both the relative abundance of bins with significant phylogenetic signal (RAsig.adj) and the mean correlation coefficient across bins (meanR) showed a peak. For ds = 0.2, and bin.size.limit = 20, in addition, we made sure that the relative contribution of stochastic processes was comparable to the phylogenetic normalized stochasticity ratio, as suggested by the authors. iCAMP was then run with these parameters using “Confidence” as the significance index (a non-parametric, one-sided confidence score) and the beta mean pairwise distance (bMPD) as the metric for phylogenetic null model analysis. One thousand randomizations were used for confidence checking. Finally, bin-level statistics were produced using bootstrapping and were output to a text file.

## Results

### The capacity for leaf bacteria to transition from soil to leaves is widespread

**Fig. 2:**
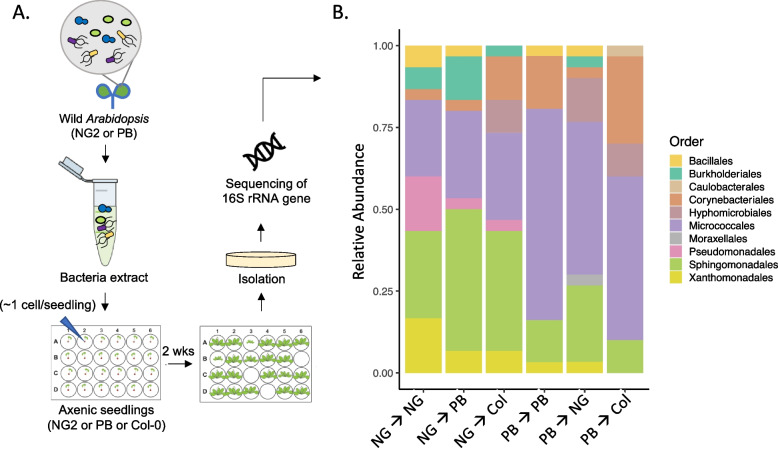
Many leaf bacteria can reach and colonize leaves when only a few cells encounter a germinating seed.** A** The in-planta isolation method based on dilution-to-extinction of leaf-associated microbes from wild *A. thaliana* leaves and inoculation onto germinating axenic seedlings, followed by 2 weeks of growth and isolation and identification of colonizers. This was used to identify leaf bacteria that are likely to be efficient colonizers when highly dilute in soil. **B** Bacteria collected from wild NG2 or PB plants were diluted to 3.5 and 1.45 cells/seedling and inoculated onto germinating NG2, PB, or Col-0 plants. Bar chart showing taxonomy of 30 random colonizers from each treatment (31 for PB PB). Taxonomy is grouped at the order level, taxonomy at the genus level and associated statistics can be found in Figure S1 and Table S2. Detailed data on numbers of plants inoculated and colonized are found in Table S1

Although soil is thought to be an important inoculum for all plant tissues, we questioned whether many leaf bacteria are likely to have the capacity to make the complex transition from soil to leaves. To answer this, we first estimated cell number distributions for strains in theoretical homogenous soils using a simple diversity model (see details in Supplementary Results). This analysis suggested that most potential leaf-colonizing bacterial strains will start the colonization process from just a few cells in the spermosphere (the area under the influence of a germinating seedling). Thus, we generated inocula from leaves from wild *A. thaliana* NG2 and PB populations, diluted them to about 3.5 and 1.45 cells/drop inoculation, respectively, and then inoculated each onto 120 axenic 3-day old NG2, PB, and Col-0 seedlings (360 plants per inoculum). Based on a test experiment, these dilutions were determined to result in sufficient colonized plants, most with only one bacterium per plant (Supplementary Results). Two weeks later, mature leaf material was collected, crushed, and plated on R2A media (Fig. 2A).

25.0% (NG2 ≥ PB) to 55.8% (PB ≥ NG2) (Table S1) of inoculated plants were colonized, suggesting that the inoculum level should result in mostly plants colonized by single bacteria. From 160 positive plants, 181 bacteria were isolated and identified. These were diverse, including 27 genera from three gram-positive (Bacillales, Corynebacterales, and Micrococcales) and seven gram-negative families (Burkholderiales, Caulobacterales, Hyphomicrobiales, Sphingomonadales, Moraxellales, Pseudomonadales, and Xanthomonadales) (Fig. 2B). One hundred forty plants (87.5%) had only one discernable colony. *Pseudomonas*, *Janthinobacterium*, and *Sphingomonas* isolates were significantly more often enriched from NG2 leaf extracts, while *Clavibacter* was more abundant from PB leaf extracts (Figure S1, Table S2). For bacteria detected often enough to statistically evaluate, there was little evidence that they preferred a particular genotype, with only *Curtobacterium* from PB slightly more frequently detected in PB plants than in other genotypes (χ^2^: *p* = 0.082, Table S2). In 20 plants (12.5%), more than one bacterium was co-isolated. For *Clavibacter*, *Methylobacterium*, and members of the Burkholderiales (*Janthinobacterium*, *Variovorax*, *Acidovorax*, and *Rugamonas*), this was significantly more often than expected (χ^2^: *p* < 0.05), suggesting they likely benefit from a partner (Table S3). Some of the Burkholderiales were only observed once, but as a group, the seven total Burkholderiales isolates (all Comamonadaceae and Oxalobacteriaceae) always were together with a co-colonizer, although this would be expected only once by chance alone (χ^2^: *p* = 2.56 × 10^−12^). Together, these findings confirm that diverse leaf-associated bacteria can efficiently transition to leaves from minute levels near a germinating seedling, suggesting that the soil-to-leaf pathway may be important for many taxa but some taxa may depend on the effects of others to make the transition.

### Early colonization of germinating seedlings shapes mature leaf bacteriomes

**Fig. 3:**
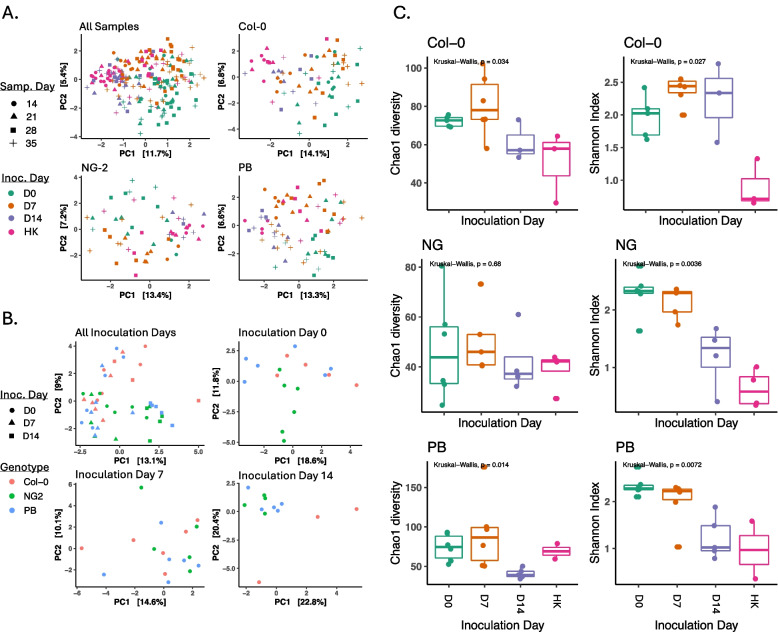
Early-inoculated plants (before the emergence of true leaves) develop distinct and diverse bacteriomes in mature leaves. Data is based on genus-level taxonomy of bacteria associated with the mature leaves of *A. thaliana* after inoculation at day 0, 7, or 14 or with heat-killed inoculum (D0, D7, D14, HK). **A** RDA ordination of beta diversity (Aitchison distance) of all samples including multiple sampling times. The results of a PERMANOVA test for the significance of factors are in Table 1. **B** RDA ordination of beta diversity (Aitchison distance), including only live-inoculated samples collected 21 days post-inoculation. The results of a PERMANOVA test for the significance of factors are in Table 2. **C** Alpha diversity (Chao1 estimates of total diversity and Shannon Index) of samples collected 21 days post

To study the importance of the soil-to-leaf transition of bacteria in a natural colonization of *Arabidopsis thaliana*, we controlled when plants of three genotypes were inoculated by a natural soil microbiota (day 0, D7, D14, or heat-killed). Whole leaf bacterial community structures were more strongly shaped by inoculation day than either sampling day (SD14, SD21, SD28, or SD35) or plant ecotype (Col-0, NG2, or PB) at all investigated taxonomic levels (ASV, genus, and order). In particular, the inoculation day effect was strongest in the Col-0 genotype (explaining up to 18% of variation at the order level) but was also significant in NG2 and PB (explaining about 10% of the variation in each) (Fig. 3A; Figures S2A and S3A; Table 1; Tables S4 and S5). The “bacteriome” of plants treated with heat-killed control inocula were much less beta- and alpha-diverse than plants treated with live inocula (Fig. 3A; Figures S2A, S3A, and S4) and generally clustered closer to plants inoculated at D14 than to other plants (Figure S4). In all genotypes, mature leaf bacteriomes (21 days post inoculation) that had been live-inoculated at D0 were more similar to those inoculated at D7 than to those inoculated at D14, especially at higher taxonomic levels (Fig. 3B; Figures S2B and S3B and between-sample beta diversity in Fig. S4). This is because many taxa were significantly enriched in early (D0 or D7)-inoculated plants compared to D14-inoculated plants (Figures S5–S7), leading to general patterns of higher alpha diversity and usually higher evenness in D0- and D7-inoculated plants (Fig. 3C; Figures S2C and S3C).

**Table 1:** Significance of factors in describing the variation in bacterial communities between samples. Values highlighted in bold have a significant p-value

All samples	Df	SumsOfSqs	MeanSqs	F.Model	*R*^2^	Pr(> *F*)
Inoculation day	3	6874.932	2291.644	5.541	**0.069**	**0.001**
Sampling day	3	4548.360	1516.120	3.666	**0.046**	**0.001**
Ecotype	2	3040.552	1520.276	3.676	**0.031**	**0.001**
Inoculation Day x Sampling Day	8	5076.620	634.578	1.534	**0.051**	**0.001**
Inoculation Day x Ecotype	6	3881.837	646.973	1.564	**0.039**	**0.001**
Sampling Day x Ecotype	6	2623.928	437.321	1.057	0.026	0.258
Inoculation Day x Sampling Day x Ecotype	15	6518.662	434.577	1.051	0.065	0.206
Residuals	162	67,004.491	413.608	NA	0.673	NA
Total	205	99,569.383	NA	NA	1.000	NA
**Col-0 samples**						
Inoculation day	3	4473.145	1491.048	3.942	**0.140**	**0.001**
Sampling day	3	2092.279	697.426	1.844	**0.066**	**0.001**
Inoculation Day x Sampling Day	8	3348.026	418.503	1.107	0.105	0.125
Residuals	58	21,936.765	378.220	NA	0.689	NA
Total	72	31,850.215	NA	NA	1.000	NA
**NG-2 samples**						
Inoculation day	3	2464.503	821.501	2.349	**0.106**	**0.001**
Sampling day	3	1869.486	623.162	1.782	**0.080**	**0.001**
Inoculation day x sampling day	8	3238.656	404.832	1.158	**0.139**	**0.043**
Residuals	45	15,736.407	349.698	NA	0.675	NA
Total	59	23,309.053	NA	NA	1.000	NA
**PB samples**						
inoculation day	3	3163.243	1054.414	2.588	**0.094**	**0.001**
sampling day	3	2753.465	917.822	2.253	**0.082**	**0.001**
inoculation day x sampling day	7	3732.816	533.259	1.309	**0.111**	**0.005**
residuals	59	24,036.932	407.406	NA	0.714	NA
Total	72	33,686.457	NA	NA	1.000	NA

The effect of plant genotypes also depended on inoculation time. Specifically, plants inoculated at D0 or D7 had more taxa that were differentially enriched between the genotypes (43 and 57 ASVs, respectively) than plants inoculated at D14 (28 ASVs, Figure S8). Additionally, 15 ASVs were shared between D0- and D7-inoculated plants, while only 3 ASVs were shared between D0- and D14-inoculated plants. However, plant genotype explained more beta diversity in D14-inoculated plants (*p* < 0.01, with 26–28% of variation depending on taxonomic level) than in plants inoculated at D0 or D7 (11–17%, insignificant at some taxonomic levels) (Table 2; Tables S6 and S7) because some differentially enriched taxa in D14-inoculated plants were highly abundant, especially in the Col-0 genotype (FiguresS9–S10). Overall, these results show that leaf bacteriome assembly is shaped uniquely by the early transition of bacteria from soil to plant leaves, resulting in higher diversity and more taxa that are influenced by plant genotypes.

**Table 2:** Significance of factors in describing the variation in bacterial communities between samples. Values highlighted in bold have a significant p-value

All	Df	SumsOfSqs	MeanSqs	F.Model	*R*^2^	Pr(> *F*)
Inoculation day	2	2743.151	1371.576	3.258	**0.126**	**0.001**
Ecotype	2	1547.307	773.654	1.838	**0.071**	**0.002**
Inoculation Day x Ecotype	4	1963.144	490.786	1.166	0.090	0.078
Residuals	37	15,574.781	420.940	NA	0.714	NA
Total	45	21,828.384	NA	NA	1.000	NA
**Inoculation day 0**						
Ecotype	2	841.835	420.918	1.456	**0.172**	**0.022**
Residuals	14	4048.468	289.176	NA	0.828	NA
Total	16	4890.303	NA	NA	1.000	NA
**Inoculation day 7**						
Ecotype	2	1074.012	537.006	1.245	**0.151**	**0.047**
Residuals	14	6040.560	431.469	NA	0.849	NA
Total	16	7114.572	NA	NA	1.000	NA
**Inoculation day 14**						
Ecotype	2	617.728	308.864	1.667	**0.270**	**0.003**
Residuals	9	1667.487	185.276	NA	0.730	NA
Total	11	2285.216	NA	NA	1.000	NA

Results are based on the Aitchison distance between samples collected at all time points at the genus level and includes plants treated with only heat-killed inoculum. Analysis was carried out with PERMANOVA with 1000 permutations.

Results are based on the Aitchison distance between samples collected at 21 DPI at the genus level and do not include the heat-killed inoculum. Analysis was carried out with PERMANOVA with 1000 permutations.

### In the overall stochastic transition of bacteria to leaves, some taxa transition deterministically

**Fig. 4:**
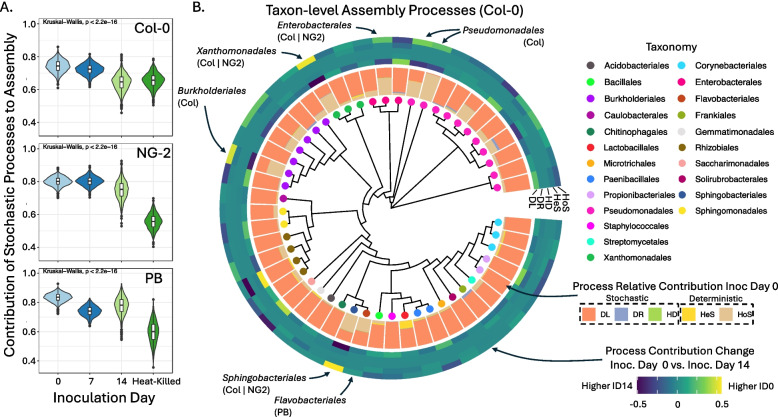
Processes shaping bacterial colonization of leaves of the *A. thaliana*genotypes Col-0, NG2, and PB depend on both inoculation time and genotype. **A **The relative importance of stochastic processes in the colonization of the leaves of plants live-inoculated at ID0, ID7, ID14, or heat-killed inoculated. Stochasticity represents the sum of dispersal limitation, homogenous dispersal, and drift, but was consistently dominated by dispersal limitation. Deterministic processes (homologous and heterologous selection), as well as an independent measure of stochasticity (pNST) are shown in Figure S10. B. Assembly processes at the level of taxonomic bins, shown for Col-0, and trees for other genotypes are shown in Figure S11. The tree is annotated with, from inside to outside: colored dots representing the majority taxonomy of each bin, A barchart showing for plants inoculated at ID0 the relative contribution of each community assembly process for each bin (DL: dispersal limitation, DR: drift, HD: homogenizing dispersal, HeS: heterogeneous selection, HoS: homogeneous selection), and A heatmap showing the change in the relative importance of each assembly process for each bin between plants inoculated at ID0 and those inoculated at ID14 (yellow means a given process is more important at ID0, dark blue is more important at ID 14). The labeled taxa are those mentioned in the text that were selected deterministically at ID0 and stochastically at ID14 (homologous selection increased by at least 20% to reach > 50% total contribution in D0). The plant genotypes where this difference was observed are labeled

To understand why plants that germinated in inoculated soil (inoculation D0) were different than those inoculated later (inoculation D14), we studied the mechanisms of assembly of leaf bacteria (stochastic vs. deterministic processes). To do so, we employed a phylogenetic bin-based null model analysis [16], which determines to what extent different colonization processes shape phylogenetically grouped bins of ASVs. Overall, stochasticity was driven by the colonization process itself, since inoculation of plants with heat-killed inoculum resulted in much less stochastic “bacteriomes” than in live-inoculated plants (Fig. 4A). In live-inoculated plants, stochastic processes dominated in all genotypes and inoculation times (> 60% contribution to assembly, Fig. 4A), with colonization slightly more stochastic in D0- than in D14-inoculated plants. Despite this dominance, deterministic processes were still important and were dominated by homogenous selection (HoS), making up ~ 15–35% relative importance depending on plant genotype (highest in Col-0, lowest in PB) and inoculation time (Figures S11A and S11B). These results were validated based on agreement with a second, independently calculated stochasticity metric, the phylogeny-based normalized stochasticity ratio [35] (Figure S11D).

At the level of phylogenetic bins, colonization processes were usually dominated by dispersal limitation (DL), a stochastic process, and this was consistent across genotypes and inoculation times (Fig. 4B). Some taxa, however, were more strongly influenced by deterministic processes. Pseudomonadales were especially strongly and consistently shaped by deterministic HoS, in particular bin Pseudomonadales 41 (including only *Pseudomonas* ASVs) (Fig. 4B; Figure S12 and detailed bin data in Supplementaryfile 1). This determinism can help explain why many outlier ASVs that were unusually abundant for their frequency (fraction of samples where they were observed) were *Pseudomonas* (Figure S13). Several other bins also colonized deterministically, particularly in D0-inoculated plants (> 50% contribution from HoS—barchart in Fig. 4B). These were Sphingobacterales 19 and Enterobacterales 38 (in Col-0 and NG2, mostly *Pedobacter* and *Enterobacter* ASVs, respectively), Xanthomonadales 35 and Burkholderiales 17 (in Col-0, mostly *Stenotrophomonas* and *Janthinobacterium*, respectively), Pseudomonadales 40 and 41 (in Col-0 and PB, mostly *Pseudomonas*), and Flavobacteriales 20 (in PB, mostly *Chryseobacterium*). Notably, all of these (except Pseudomonadales 41 and 49 in NG2 and Col-0) were only deterministic in D0-inoculated plants but colonized stochastically in D14-inoculated plants (the difference between D0 and D14 was > 25%) (heatmap in Fig. 4B, Figure S12 and Supplementary file 1). For several of these same bins, HoS was more important at D0 than D14 in other genotypes as well but did not reach > 50% at D0 (Burkholderiales 17 and Flavobacteriales 20 in NG2 and Enterobacterales 38 in PB). Together, these results show that after seedling germination in soil, the transition from soil to mature leaves is a stochastic process for many colonizers. Most taxa that do make this transition deterministically, however, do so only during this complex transition and not when they are added later, helping to explain why plants colonized naturally from soil assemble distinct leaf bacteriomes.

### Plant–microbe interactions shape the colonization of deterministic taxa

Determinic colonization indicates that a particular bacterium is not easily replaced, a sign that it could occupy a specialized niche. Thus, we reasoned that treatments that strongly affect plant phenotypes and physiology would most strongly affect deterministic colonizers. To test this, we selected *Pseudomonas viridiflava* 3D9, a strain isolated in the *in-planta* isolation experiment (Fig. 1B, treatment NG->NG). *Pv*3D9 is an opportunistic pathogen native to the NG2 genotype—just a few cells inoculated onto axenic NG2 seedlings are enough to nearly always kill the host plant (Figure S14). It was isolated from healthy plant tissue, however, and when we amend it to natural soil, we consistently observe that its presence actually rescues plant growth (Figure S15). This could be because opportunistic *P. viridiflava* strains interact with plant immunity [36], which in turn could protect against detrimental soil microbes. Consistent with this, we observe a slightly stronger immune response (ROS burst in response to the flg22 peptide) in some *Pv*3D9-treated plants (Figure S15). Thus, in a natural soil, *Pv*3D9 affects plant physiology and phenotypes, and so, we could use it to test the hypothesis that this should poise it to mainly affect deterministic colonizers.

**Fig. 5:**
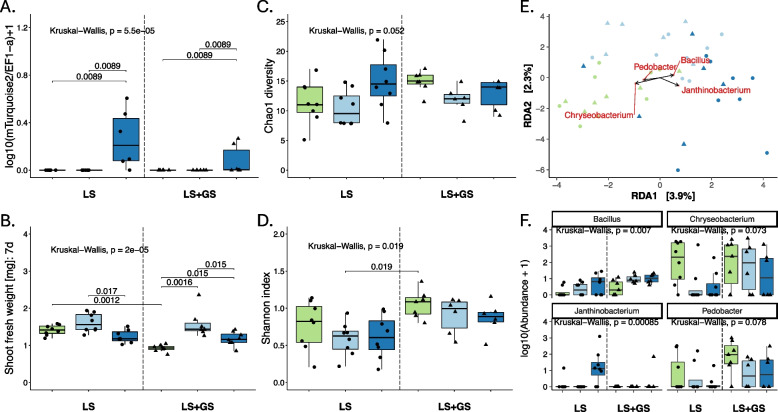
Bacteria that deterministically transition from soil to mature leaves are influenced by other colonizers. Throughout, green corresponds to the mock treatment (no 3D9-141), light blue to the low 3D9-141 soil amendment, and dark blue to the moderate 3D9-141 soil amendment. **A***Pseudomonas viridiflava* 3D9-141 abundance in mature leaf tissue based on qPCR measurement of copies of a vector-inserted gene for the mTurquoise2 protein normalized to the host plant elongation factor 1. **B** The fresh weight of *A. thaliana* shoots 7-days post-germination. **C**, **D** Alpha diversity of bacterial genera in mature leaf tissues (14 days post-germination) assessed by Chao1 metric (total estimated diversity) and Shannon index (diversity considering evenness of distribution of taxa). In **A**–**D**, a Kruskal–Wallis test was performed on the data overall, and when it was < 0.05, a Wilcoxon test was used to evaluate differences between groups. Signficant *p*-values are shown. **E** Principal components analysis of Aitchison distance between samples, constrained for treatment, showing biplot arrows for the top 3% of bacteria that correlate to the two axes. Circles correspond to LS and triangles to LS + GS, as in the boxplots. **F** Boxplots showing the abundance of the bacteria identified using the biplot analysis in **E** with *p*-value result of a Kruskal–Wallis test for significant differences between groups

As previously observed (Figure S15), plants grown in laboratory soil mixed with a natural garden soil slurry (LS + GS) showed about 35% decreased growth compared to laboratory soil alone (LS) (Fig. 5B). *Pv*3D9-141 (a labeled version of *Pv*3D9 detectable via qPCR and selectable markers [25]) reversed this, increasing the leaf fresh weight in a low amendment (~ 0.8 cells in the initial spermosphere) back to LS levels. In a moderate amendment (~ 800 cells in the initial spermosphere), shoot fresh weight was less than the low treatment, but still higher than mock levels (Fig. 5B). 3D9-141 reached mature leaves in the moderate treatment as measured by both qPCR (Fig. 5A) and resistant, fluorescent CFUs (Figure S16), but not in the low treatment. Based on total CFU counts, 3D9-141 made up roughly 1% of the leaf bacteriome and its median level in leaves was not significantly affected by the addition of GS. Thus, the opportunistic pathogen 3D9-141 can transition to plants and leaves depending on its inoculation level, and in natural soil, it always had a significant, positive effect on plant phenotypes.

Next, we tested how 3D9-141 influenced the colonization of other bacteria in whole-leaf samples. Based on CFU counts, there was no clear effect of the soil treatment or 3D9-141 on total leaf bacterial loads (Figure S16). The addition of garden soil did not significantly increase the bacterial diversity in mature leaves, but it did increase the evenness of the community (Fig. 5C, D). 3D9-141 appeared to slightly affect the medians of both alpha diversity and evenness, but it was not significant with conservative non-parametric statistics. It did, however, have a significant effect on bacterial community structure, with the effect of the soil type and 3D9-141 treatments each independently explaining about 6–10% of variation depending on the taxonomic level (Table 3). Generally, low and moderate 3D9-141 treatments clustered separately, driven by distinct changes to several taxa. Pseudomonas itself was only strongly correlated to treatments at the ASV level, while other taxa were clearly affected by treatments as well (Fig. 5E; Figures S17 and S18). Two of the four genera most strongly affected by 3D9-141 were affected similarly in both soils: *Bacillus* (Bacillales), whose abundance always increased with 3D9-141 treatment and *Pedobacter* (Sphingobacterales), which tended to be suppressed by 3D9-141. On the other hand, *Chryseobacterium* (Flavobacterales) was strongly suppressed by 3D9-141 at both low and moderate levels mainly in LS, while *Janthinobacterium* (Burkholderiales) was much higher abundance in LS only in the moderate treatment, when 3D9-141 reached leaves, but was hardly detected at all in LS + GS (Fig. 5F). Similar patterns at the ASV and order level underscore that effects were highly taxa-specific and depended on both soil and 3D9-141 inoculation level (Figures S17 and S18). Notably, three of four genera affected by 3D9-141 (*Chryseobacterium*, *Janthinobacterium*, and *Pedobacter*) were also strongly shaped by homogeneous selection in the natural colonization of NG2 plants (Fig. 4). Thus, taxa that behave deterministically in natural colonization appear to also be those that are most responsive to the effects of soil amendment with 3D9-141.

**Table 3:** Significance of factors in describing the variation in bacterial communities between samples. Values highlighted in bold have a significant p-value

Factor (PERMANOVA ASV level)	Df	SumsOfSqs	MeanSqs	F.Model	*R*^2^	Pr(> *F*)
3D9-141 treatment (mock, low, moderate)	2	1507.641	753.820	1.593	**0.068**	**0.004**
Soil type (LS/LS + GS)	1	2138.834	2138.834	4.519	**0.097**	**0.001**
Treatment x soil	2	971.754	485.877	1.027	0.044	0.394
Residuals	37	17,511.574	473.286	NA	0.791	NA
Total	42	22,129.803	NA	NA	1	NA
**Factor (PERMANOVA genus level)**	**Df**	**SumsOfSqs**	**MeanSqs**	**F.Model**	***R***^**2**^	**Pr(> *F*)**
3D9-141 treatment (mock, low, moderate)	2	372.982	186.491	1.363	**0.062**	**0.075**
Soil type (LS/LS + GS)	1	368.900	368.900	2.695	**0.061**	**0.002**
Treatment x soil	2	245.005	122.503	0.895	0.040	0.648
Residuals	37	5064.163	136.869	NA	0.837	NA
Total	42	6051.050	NA	NA	1	NA
**Factor (PERMANOVA order level)**	**Df**	**SumsOfSqs**	**MeanSqs**	**F.Model**	***R***^**2**^	**Pr(> *F*)**
3D9-141 treatment (mock, low, moderate)	2	265.792	132.896	1.771	**0.078**	**0.008**
Soil type (LS/LS + GS)	1	224.703	224.703	2.995	**0.066**	**0.001**
Treatment x soil	2	128.486	64.243	0.856	0.038	0.683
Residuals	37	2775.851	75.023	NA	0.818	NA
Total	42	3394.831	NA	NA	1	NA

## Discussion

### The transition of bacteria from soil to phyllosphere is an important colonization route

Bacteriomes that develop in mature leaves after seedling germination have important implications for host plant health [37]. The goal of this work was to gain better insight into how the well-known effects of host and environmental factors [6] shape natural colonization processes in these important communities. Leaf bacteriomes are composed of cells that must arrive in one of two ways: transitioning following seed germination, either from soil or from cells attached to or in seeds that were transmitted vertically from parent plants, or later arrival and “invasion” of already emerged leaves. Transitioning from soil is thought to be a very important route since leaf bacteria are typically mostly composed of a subset of the taxa found in soils [6]. The importance of the “soil route” is also recognized in commercial applications, where seed impregnation with biological treatments is meant to improve the colonization success of beneficial microorganisms [38]. However, this route is complex: bacteria must find and reach germinating seedlings and ultimately multiply on or in the growing plant to eventually establish in true leaves that only emerge later. Additionally, our simple model of soil bacterial diversity illustrated the complication that there will usually just be a few chances for success since most strains will start from just a handful of cells in the vicinity of the seedling. To our knowledge, there is little known about which bacteria efficiently manage this complex task in a natural context, how they do so, and what influences them. However, we here experimentally showed that many bacteria can and do manage this transition and that it leaves distinct signatures in the leaf bacteriome.

### Natural colonization results in uniquely diverse leaf bacteriomes

In the transition to leaves from a diverse natural soil, we found that most bacterial colonizers exhibited stochastic assembly patterns. In neutral theory, stochasticity indicates that the differences in fitness between taxa are not significant, making their distribution largely subject to neutral processes including dispersal limitation, homogenizing dispersal, and drift [16]. Thus, while many bacteria can reach and colonize leaves, most apparently do so with similar fitness. This can enable simultaneous transitioning from the soil of many taxa that are essentially exchangeable and can help explain the unique and high diversity of taxa in naturally colonized plant leaves (inoculation day 0 in this study). It should be noted that stochasticity in this context refers only to niche fitness *differences* among leaf-colonizing bacteria and does not imply that leaf colonization is just a random selection of environmental bacteria. On the contrary, leaf bacterial diversity is usually far less than soil bacterial diversity, indicating that “phyllosphere-associated bacteria” are different than other soil bacteria that do not make the transition [39]. Supporting this, in our 1-on-1 plant-bacteria enrichment experiment, a wide diversity of typical leaf bacteria could start from just a few cells near a germinating seedling and weeks later reach detectable levels in mature leaves. Thus, these taxa are apparently well-adapted to thrive on primary resources from host plants and to deal with the harsh conditions in the leaf environment, even if they do so stochastically. Host physiology including plant immunity, as well as primary resources that stochastic taxa depend on including plant exudates, will be variable across host species and environments [40]. Thus, an approach like what we have used here can help identify these efficient colonizers. This would be important for applications, to know which taxa will thrive when given an advantage, for example, via seed impregnation or soil amendments.

At least two factors could explain the stochastic recruitment of many or most leaf bacteria. For one, many leaf colonizers could use similar resources and have similar fitness so that none drastically outcompete others. Resource overlap, however, was previously found to be a relatively poor predictor of colonizer success on leaves [41]. On the other hand, resource overlap is usually calculated from resource preferences from metabolic models or in vitro experiments because of the difficulty of measuring the activity of bacteria *in-planta*. Indeed, we still know very little about niches during host colonization [42] and it is possible that there is extensive overlap among commensal bacteria. A second possibility is that space is limited and occupied on a “first-come-first-served” basis. This is supported by various studies showing that bacteria colonizing healthy leaves are limited spatially, for example, to epidermal cracks [43] or microscopic droplets [44] and that colonizers that establish there tend to be stable to subsequent invasion [23]. Regardless, the process of transitioning from soil to mature leaves is complex and may involve bacteria transitioning between multiple niches before reaching leaves. Thus, to understand and manipulate it, improved techniques to understand niches and niche diversity of non-pathogenic bacteria *in-planta* will be required.

### The emergence of determinism in the midst of stochasticity

Our results suggest that most leaf-colonizing bacteria transitioned from soil to leaves stochastically. However, a few taxa showed strong signs of determinism (specifically homogeneous selection). It is not surprising that several *Pseudomonas* bins consistently colonized deterministically, given the well-described adaptation of *Pseudomonas* to *A. thaliana* colonization [45]. On the other hand, bins dominated by *Pedobacter*,* Enterobacter*,* Stenotrophomonas*,* Janthinobacterium*, and *Chryseobacterium* also colonized deterministically. In contrast to stochastic selection, which occurs when multiple taxa can occupy a niche with similar fitness (i.e., are exchangeable), homogenous selection implies that the taxa likely have more specialized niches [16], which likely also are shaped by plant exudates. Supporting this, we previously found that several taxa, including Burkholderiales taxa in the families Comamonadaceae and Oxalobacteraceae (like *Janthinobacterium*), are strongly enriched in some *A. thaliana* genotypes due to aliphatic glucosinolates, specialized metabolites of *A. thaliana*. We were able to identify a specialized metabolic niche whereby only certain bacteria with myrosinase enzymes (including an Enterobacterales) can metabolize glucosinolates as a carbon source, and specific cross-feeding interactions support the enrichment of other taxa (*Pseudomonas* and perhaps *Janthinobacterium*) [14]. A similar phenomenon was recently observed in maize, where specialized lactonases enable specific bacteria to grow on benzoxazinoids, leading to rhizosphere enrichment [46]. Given the wide diversity of specialized metabolites that plants produce [47], we speculate that these can be the basis of many specialized niches that underly deterministic recruitment in plants. However, new approaches are needed to understand what is available to colonizers and how bacteria take advantage of them. The approach we use here to identify deterministic taxa may be a good way to identify those whose colonization niche should be studied more closely.

### Deterministic processes in natural colonization rely on the soil-to-leaf transition

Notably, here we found that except for *Pseudomonas*, all the taxa that were deterministic in the transition from soil to leaves colonized stochastically when soil inoculum was applied directly onto leaves (inoculation day 14). It is currently unclear why deterministic niche establishment in leaves would be dependent on at least some part of the complex transition from soil. However, several explanations are plausible. First, germinating seedlings exude a broad mix of compounds into the soil matrix that shifts in composition over time [48]. Exudates can serve as signals that cause soil bacteria to alter their phenotypes to promote host colonization [49]. Therefore, exudates secreted early on could serve as a signal to activate the metabolic pathways required to establish a specialized niche. Second, plants use molecular recognition machinery to react specifically to different types of microbes [50], where responses include altered secondary metabolism. Thus, the makeup of the soil microbiome at early colonization timepoints, by altering plant physiology and chemistry, can lead to altered patterns of deterministic colonization. This is consistent with our finding that even very low-level natural soil amendment with *Pv*3D9, an opportunistic pathogen, promoted plant growth, likely indicating that it altered the plant’s immune status. These changes to plant physiology had either direct effects or effects that rippled into the leaf bacteriome, but these were almost strictly limited to deterministically colonizing taxa, suggesting that processes they depend on had been altered.

A third possibility could be that important interactions between deterministic taxa developed during early colonization. Our results strongly suggest that deterministic colonization of leaves by some taxa arises out of inter-bacterial interactions. In the *in-planta* isolation experiments, *Oxalobacteraceae* and *Comamonadaceae* bacteria were always only found with a partner bacterium. Consistently, our previous work showed that *Oxalobacteraceae* and *Comamonadaceae* colonization in *A. thaliana* leaves was linked to aliphatic glucosinolates, but in vitro tested isolates probably relied on another bacterium that could metabolize the compound [14]. Similarly, in an enrichment of bacteria from peppers on the secondary metabolite capsaicin, two partners (a *Pseudomonas* and the Comamonadaceae *Variovorax*) fully relied on one another to use it as the sole carbon and nitrogen source [51]. More broadly, Comamonadaceae and Oxalobacteraceae have been highlighted as being strongly positively correlated to a range of bacteria in wild *A. thaliana*, including links to both Sphingobacteriaceae and Flavobacteriacae (which include *Pedobacter* and *Chryseobacter*ium, taxa found here to colonize deterministically with *Janthinobacterium*) [52, 53]. Thus, we hypothesize that these bacteria enter into diverse interactions with other bacteria that define their niche and promote deterministic colonization. Regardless, once taxa are identified that colonize deterministically in a given environment, an important next step will be elucidating the molecular underpinning of the colonization mechanisms to realize targeted manipulations.

## Conclusions

Understanding how plant-associated bacteria assemble in tissues like leaves is a critical step toward harnessing their potential for sustainable plant protection. While soil clearly is an important inoculum of bacteria for leaf tissues, the complex transition of soil bacteria to leaves following seedling germination has largely remained a black box. This work demonstrated that the early stages following germination are critical for the establishment of diverse bacteriomes in mature leaves. This is because of the unique mix of stochastic and deterministic mechanisms that arises during early colonization to shape individual bacterial taxa. Deterministic mechanisms especially are shaped by both host genotype and host-microbe interactions. A more detailed understanding of how these factors play out will still require characterizing the pathway of transition in or on plants. Thus, characterizing the mechanisms shaping bacterial taxa offers a way forward for targeted manipulation and optimization of leaf-associated bacteriomes.

## Supplementary Information

Additional file 1.

Additional file 2. Figure S1. Genus-level taxonomy and number of isolates of each genus recovered from in-planta isolation, where NG or PB-derived bacteria were inoculated onto NG, PB or Col-0 genotypes. Associated statistics can be found in Table S2.
Figure S2. Early-inoculated plants (before the emergence of true leaves) develop distinct and diverse bacteriomes in mature leaves. Data is based on ASVs associated with the mature leaves of A. thaliana after inoculation at day 0, 7 or 14 or with heat-killed inoculum (D0, D7, D14, HK). A. RDA ordination of beta diversity (aitchison distance) of all samples including multiple sampling times. Results of a PERMANOVA test for significance of factors is in Table 1. B. RDA ordination of beta diversity (aitchison distance), including only live-inoculated samples collected 21 days post inoculation. Results of a PERMANOVA test for significance of factors is in Table 2. C. Alpha diversity (Chao1 estimates of total diversity and Shannon Index) of samples collected 21 days post inoculation.
Figure S3. Early-inoculated plants (before the emergence of true leaves) develop distinct and diverse bacteriomes in mature leaves. Data is based on Order-level taxonomy of bacteria associated with the mature leaves of A. thaliana after inoculation at day 0, 7 or 14 or with heat-killed inoculum (D0, D7, D14, HK). A. RDA ordination of beta diversity (aitchison distance) of all samples including multiple sampling times. Results of a PERMANOVA test for significance of factors is in Table 1. B. RDA ordination of beta diversity (aitchison distance), including only live-inoculated samples collected 21 days post inoculation. Results of a PERMANOVA test for significance of factors is in Table 2. C. Alpha diversity (Chao1 estimates of total diversity and Shannon Index) of samples collected 21 days post inoculation.
Figure S4. Between-sample beta diversity (Aitchison distances) comparing bacterial communities associated with mature A. thaliana leaf samples (21 DPI) at the ASV (left), Genus (middle) and Order (right) levels. Within-group distances (D0_D0, D7_D7 or D14_D14) show the variation in the bacterial community structure in a given treatment group. Between-group distances (D7_D0, D14_D0 and D14_D7) show how similar or different samples are between two treatments. In all cases, the stars show sinificant differences compared to the D0_D0 group.
Figure S5. Bacterial taxa (ASVs) in mature leaf samples (combined SD21, 28, 35) that were differentially enriched between plants inoculated at D0 and D14. In the boxplots only the top 5 ASVs are shown (smallest fdr-adjusted p-values) because of space constraints. A. in Col-0, B. in NG-2, C. in PB. D. The total number of ASVs that were differentially enriched in each genotype and their overlap. Total detections are based on a DESeq2 analysis with p < 0.001. Kruskall-wallis p-values are additionally shown for each plot.
Figure S6. Bacterial taxa (Genera) in mature leaf samples (combined SD21, 28, 35) that were differentially enriched between plants inoculated at D0 and D14. In the boxplots only the top 5 genera are shown (smallest fdr-adjusted p-values) because of space constraints. A. in Col-0, B. in NG-2, C. in PB. D. The total number of genera that were differentially enriched in each genotype and their overlap. Total detections are based on a DESeq2 analysis with p < 0.001. Kruskall-wallis p-values are additionally shown for each plot.
Figure S7. Bacterial taxa (Orders) in mature leaf samples (combined SD21, 28, 35) that were differentially enriched between plants inoculated at D0 and D14. In the boxplots only the top 5 Orders are shown (smallest fdr-adjusted p-values) because of space constraints. A. in Col-0, B. in NG-2, C. in PB. D. The total number of taxa that were differentially enriched in each genotype and their overlap. Total detections are based on a DESeq2 analysis with p < 0.001. Kruskall-wallis p-values are additionally shown for each plot.
Figure S8. Total number of taxa correlated to ecotype in mature plants (combined SD21, 28, 35) that had been inoculated at D0, D7 or D14. A. ASV-level taxa, B. Genus-level taxa, and C. Order-level taxa. Detections were based on DESeq analysis with p < 0.01.
Figure S9. Bacterial taxa that were differentially enriched between ecotypes in mature leaf samples (combined SD21, 28, 35) in plants inoculated at D0. A. ASV-level taxa, B. Genus-level taxa, and C. Order-level taxa. Detections are based on DESeq analysis with p < 0.01, but only the top 5 taxa are shown because of space constraints. The total number of differentially enriched taxa are shown in Figure S8. Kruskall-wallis p-values are additionally shown for each plot.
Figure S10. Bacterial taxa that were differentially enriched between ecotypes in mature leaf samples (combined SD21, 28, 35) in plants inoculated at D14. A. ASV-level taxa, B. Genus-level taxa, and C. Order-level taxa. Detections are based on DESeq analysis with p < 0.01, but only the top 5 taxa are shown because of space constraints. The total number of differentially enriched taxa are shown in Figure S8. Kruskall-wallis p-values are additionally shown for each plot.
Figure S11. Processes shaping bacterial colonization of leaves of the A. thaliana genotypes Col-0, NG2 and PB depend on both inoculation time and genotype. A.–C. are based on the phylogenetic bin-based null model analysis in iCAMP. A.-B. The relative importance of deterministic processes (homogeneous and heterogeneous selection, respectively) and C. the relative importance of of stochastic processes (the sum of Dispersal limitation, Drift, and Homogenizing dispersal) in colonization of the leaves of plants inoculated at ID0, ID7, ID14 or never inoculated. D. The phylogenetic normalized stochasticity ratio (phylogenetic NST) is also provided as an independent measure of stochasticity for comparison. The top row is Col-0, the second row is NG-2 and the third row is PB.
Figure S12. Processes shaping bacterial colonization of leaves of the A. thaliana genotypes Col-0, NG2 and PB depend on both inoculation time and genotype. Assembly processes at the level of taxonomic bins. The trees are all three identical and are annotated with, from inside to outside: Colored dots representing the majority taxonomy of each bin, A barchart showing for plants inoculated at ID0 the relative contribution of each community assembly process for each bin (DL: Dispersal limitation, DR: Drift, HD: Homogenizing dispersal, HeS: Heterogeneous selection, HoS: Homogeneous selection), and A heatmap showing the change in relative importance of each assembly process for each bin between plants inoculated at ID0 and those inoculated at ID14 (yellow is higher at ID0 dark blue is higher at ID 14). Figure 3B in the main text also shows labeled the taxa that are mentioned in the text that were selected deterministically at ID0 and stochastically at ID14 (homologous selection increased by at least 20% to reach >50% total contribution in D0).
Figure S13. Correlation of the log mean abundance of ASVs to either the coefficient of variation (a measure of stochasticity of abundance) or the fraction of samples the taxa was detected in. Measurements are shown for ecotypes Col-0 (A), NG2 (B) and PB (C). The measures are calculated using data from leaf samples collected at SD 21, 28 and 35.
Figure S14. Pseudomonas viridiflava 3D9 is an opportunistic pathogen of A. thaliana. Germinating A. thaliana Col-0 or NG2 seedlings were inoculated with very low levels of three bacteria (CFU/seedling is indicated). After 14 days, plant phenotypes were recorded. healthy = no symptoms, stressed = purple/discolored or chlorotic leaves, necrotic = spots of dead tissue on at least one leaf, or dead. N=10.
Figure S15. Pseudomonas viridiflava 3D9 mixed into a natural soil promotes growth and alters the immune response of A. thaliana. A. The fresh weight of A. thaliana shoots 25-days post-germination. N= 5-9. B. The response of A. thaliana leaf tissue to bacterial flg22 peptide as measured in a luminescence assay for reactive oxygen species (ROS) production. The total ROS burst is presented as the area under the luminescence curve (N=6). P-values represent the results of a 1-sided t-test to evaluate if there was an increase in plant weight or ROS response of Peat+Soil vs 3D9 treatments.
Figure S16. Pseudomonas viridiflava 3D9 transitions from soil to leaves based on fluorescent cell counts. Seeds were germinated in standard laboratory soil (LS) or LS amended with an extract of a natural garden soil (LS+GS). The three treatments in each soil are no Pseudomonas viridiflava 3D9, low 3D9 (~1000 cells/cm3) or moderate 3D9 (~1x106 cells/cm3). 14-d old plants were harvested and bacterial CFU counts were made on LB medium with nystatin (LB+N – total bacterial count), LB+N with gentamycin, chloramphenicol and bacitracin (LB+NGCB – selection against gram + bacteria) or LB+N with bacitracin and boric acid (LB+NBba – selection of Pseudomonads). The same plates were also observed for fluorescent colonies with a stereo microscope equipped with a GFP filter to estimate CFUs expressing mTurquoise2 (Pseudomonas viridiflava 3D9).
Figure S17. Bacteria that deterministically transition from soil to mature leaves are influenced by P. viridiflava 3D9 soil amendment (ASV level analysis to complement Figure 5). (A. and B.) Alpha diversity of bacteria in mature leaf tissues (14 days post germination) assessed by Chao1 metric (total estimated diversity) and Shannon index (diversity considering evenness of distribution of taxa). A Kruskal-Wallis test was performed on the data overall and when this was significant a Wilcoxon test was used to evaluate differences between groups. Significant p-values are shown. (C.) Principal components analysis of Aitchison distance between samples, constrained for treatment, showing biplot arrows for the top 1% of ASVs that correlate to the two axes. (D.) Boxplots showing the abundance of the bacteria identified using the biplot analysis in (C.) with p-value result of a Kruskal-Wallis test for significant differences between groups.
Figure S18. Bacteria that deterministically transition from soil to mature leaves are influenced by P. viridiflava 3D9 soil amendment (Order-level analysis to complement Figure 5). (A. and B.) Alpha diversity of bacteria in mature leaf tissues (14 days post germination) assessed by Chao1 metric (total estimated diversity) and Shannon index (diversity considering evenness of distribution of taxa). A Kruskal-Wallis test was performed on the data overall and when this was significant a Wilcoxon test was used to evaluate differences between groups. Significant p-values are shown. (C.) Principal components analysis of Aitchison distance between samples, constrained for treatment, showing biplot arrows for the top 3% of taxa that correlate to the two axes. (D.) Boxplots showing the abundance of the bacteria identified using the biplot analysis in (C.) with p-value result of a Kruskal-Wallis test for significant differences between groups.
Figure S19. Preston’s Octave plots for the number of strains (y-axis) that fall within a range for a given number of cells per strain (x-axis). The three plots correspond to three orders of magnitude of N, the density of bacterial cells in soil (cells/mm3) and each plot is calculated for three levels of alpha, Fisher’s diversity index. The equations that the plots are based on can be found in the supplementary results.
Figure S20. Results of test experiment to identify leaf bacteria that can reach and colonize leaves when only a few cells encounter a germinating seed. Bacteria collected from wild NG2 plants were diluted to near extinction (0-1 cells/plant) and inoculated onto liquid or solid R2 medium or onto germinating seedlings. The bar chart shows the taxonomy of randomly picked isolates from the medium and from mature leaves where colonization was detected by PCR.
Table S1: Number of bacterial colonizers recovered by traditional medium-based isolation methods in comparison with our in-planta approach (2018) and comparison between cross-inoculations of leaf inocula onto different plant genotypes (2019).
Table S2: fdr-adjusted p-values for genus-level taxonomy and number of isolates of each genus recovered from in-planta isolation, where NG or PB-derived bacteria were inoculated onto NG, PB or Col-0 genotypes. P-values are based on a X2 test to determine whether a genus significantly more often originated from PB or NG (origin) or was recovered significantly more often in PB, Col or NG. p-values are only shown for genera where there were at least two observations in the tested conditions.
Table S3: P-values for the likelihood that the number of times a genus was observed co-colonizing with another taxa was by chance alone. P-values are based on a C2 test comparing the expected number of observations (87.5% of total observations alone and 12.5% of total observations with a co-colonizer – based on the totals in the experiment) vs. the actual number of times the genus was observed alone and with a co-colonizer. Genera in bold are those who were mentioned in the text because they were found more often paired with another bacterium than alone. Orange colored genera denote the Burkholderiales.
Table S4: Significance of factors in describing the variation in bacterial communities between samples. Results are based on the Aitchison distance between samples collected at all time points at the level of ASVs. Analysis was carried out with PERMANOVA with 1000 permutations.
Table S5: Significance of factors in describing the variation in bacterial communities between samples. Results are based on the Aitchison distance between samples collected at all time points at the Order level. Analysis was carried out with PERMANOVA with 1000 permutations.
Table S6: Significance of factors in describing the variation in bacterial communities between samples. Results are based on the Aitchison distance between samples collected at 21 DPI at the ASV level. Analysis was carried out with PERMANOVA with 1000 permutations.
Table S7: Significance of factors in describing the variation in bacterial communities between samples. Results are based on the Aitchison distance between samples collected at 21 DPI at the Order level. Analysis was carried out with PERMANOVA with 1000 permutations.
Table S8: Counts and p-values for the likelihood that the number of times a genus was observed in-planta is the same as the expected  number of observations based on counts in R2A medium. P-values are based on a C2 test comparing the expected number of observations (calculated based on the frequency observed in R2A medium) vs. the actual number of observations.

## Data Availability

The 16S rRNA sequencing datasets generated and analysed during the current study are available in the NCBI SRA repository BioProject number PRJNA1174406, https://www.ncbi.nlm.nih.gov/bioproject/PRJNA1174406. Scripts used to analyze the data in R to create the figures and tables, as well as the raw data tables and metadata files will all be made publicly available before publication on Figshare: 10.6084/m9.figshare.27290064.v1.
